# Modification Mechanism and Rheological Properties of Emulsified Asphalt Evaporative Residues Reinforced by Coupling-Modified Fiber

**DOI:** 10.3390/ma14237363

**Published:** 2021-11-30

**Authors:** Zhaofeng Lu, Lin Kong, Zhaoyi He, Hao Xu, Kang Yang, Zuzhen Shen, Zhaodong Huang

**Affiliations:** 1 School of Mechanotronics and Vehicle Engineering, Chongqing Jiaotong University, Chongqing 400074, China; 2 School of Civil Engineering, Chongqing Jiaotong University, Chongqing 400074, China; konglin@mails.cqjtu.edu.cn (L.K.); 611210111004@mails.cqjtu.edu.cn (H.X.); yangkang0125@163.com (K.Y.); S54846302@163.com (Z.S.); h495141839@163.com (Z.H.); 3 School of Traffic & Transportation, Chongqing Jiaotong University, Chongqing 400074, China

**Keywords:** emulsified asphalt, silane coupling agent, basalt fiber, rheological properties, modification mechanism

## Abstract

In order to solve the problems of the smooth surface of basalt fiber and its weak interfacial adhesion with emulsified asphalt cold recycled mixture, a silane coupling agent (KH550) was used to treat the surface of basalt fiber and the effects of treatment concentration and soaking time on fiber modification were studied. The influence of silane coupling-modified basalt fiber (MBF) on the rheological properties of emulsified asphalt evaporation residue was studied at high and low temperatures using three routine index tests: a dynamic shear rheological test (DSR), a bending beam rheological test (BBR), and a force ductility test. The elemental changes of the fiber before and after modification and the microstructure of the emulsified asphalt evaporation residue with the coupling-modified fiber were analyzed by Fourier infrared spectroscopy (FT-IR), scanning electron microscopy (SEM), and X-ray energy dispersive spectroscopy (EDS), which is used to study the modification mechanism of emulsified asphalt evaporation residue reinforced by coupling-modified fiber. The results indicate that the concentration and soaking time of the silane coupling agent have a great influence on the surface morphology and mechanical properties of the fiber, and that the optimal treatment concentration is 1.0% and the optimal soaking time is 60 min. The addition of coupling-modified fibers can reduce the phase angle and unrecoverable creep compliance of emulsified asphalt evaporation residue, increase the rutting factor and creep recovery rate, and improve the elastic recovery ability and permanent deformation resistance. However, excessive fiber will weaken the ductility of emulsified asphalt at low temperatures. The appropriate content of silane coupling-modified fiber (MBF) is 1.5%. After silane coupling modification, the fiber surface becomes rough and cohesion is enhanced between the fiber and the emulsified asphalt base. Silane coupling-modified basalt fiber (MBF) acts as reinforcement for stability and bridging cracks.

## 1. Introduction

The cold recycling technology of emulsified asphalt plant mix is energy saving and environmentally friendly, and can utilize recycled asphalt pavement (RAP) materials in large quantities, so it is a research hotspot in the field of road engineering at home and abroad [1,2,3,4]. Emulsified asphalt cold recycled mixture has problems such as low initial strength, poor crack resistance after molding, and insufficient water stability. It is mostly used for pavement base courses or lower layers of low-grade highways [5]. The performance of emulsified asphalt evaporation residue affects the performance of emulsified asphalt cold recycled mixture. In order to further improve the application level and road performance of the emulsified asphalt cold recycled mixture, it is necessary to improve the comprehensive performance of the emulsified asphalt evaporation residue [6].

Basalt fiber (BF) has the advantages of high modulus, good impact resistance, excellent alkali and acid resistance, high durability, etc., which makes it commonly used in asphalt concrete and cement concrete [7,8,9,10,11]. A large number of scholars have improved emulsified asphalt performance by adding basalt fiber (BF) [12,13,14,15]. Emulsified asphalt has the disadvantages of long demulsification time, slow formation of the strength of the mixture, and poor initial strength of the mixture. Basalt fiber can significantly improve the high temperature stability, low temperature crack resistance, freeze-thaw resistance, and water damage resistance of the emulsified asphalt mixture. Emulsified asphalt mixture is a multi-level spatial network structure dispersion system, and the fibers are finely dispersed in the emulsified asphalt residue. When the fiber-reinforced material receives external stress, the fiber can delay the germination of micro-cracks and avoid the further development of macro-cracks [16]. Due to the bridging effect of the fibers, the fiber reinforced material changes from brittle fractures to ductile fractures, which form a stress buffer zone. Although the mechanical properties of the fiber itself are very important, the degree of interfacial bonding of fiber composites plays a key role in toughening [17]. A strong interface bonding effect is achieved between the fiber and the matrix, which can avoid debonding, pulling out, and fiber sliding at the interface [18].

However, basalt fiber (BF) is under a smooth surface and has poor adhesion, and an insufficient enhancement effect on the performance of emulsified asphalt [19,20]. The adhesion and wettability of basalt fiber and emulsified asphalt are poor, which makes the fiber emulsified asphalt mixture unable to fully play to the advantages of the mechanical properties. The surface modification of fibers can improve the exchange between fibers and matrix materials, improve the wettability of basalt fibers, form new chemical bonds with the matrix, and ultimately improve the performance of fiber composites. Silane coupling agent, as an organosilicon compound with two groups at the same time, can tightly connect two types of substances with different properties and is of great significance to material surface modification and material bonding, for making the combination of BF and emulsified asphalt closer, as well as ultimately improving the interfacial bonding performance of BF and emulsified asphalt [21,22]. M. Iorio et al. [23] modified the BF surface to improve the basalt/cement interface in cement-based composites. Studies have revealed that polymer grafted to the surface of the fiber can enhance the bonding force of the cement interface. Deak [24] found that the use of silane coupling agents can improve the interfacial bonding properties of basalt fibers and composite materials. The effect of 3-glycyloxypropyltrimethoxysilane is the best. Maria [25] studied the effect of basalt fiber in asphalt mixture. Studies have found that basalt fibers can enhance the crack resistance of asphalt mixtures. Song [26] found that the fiber surface after silane coupling agent treatment became rougher. The dispersion of fiber is good and the breaking strength is enhanced. The BF could be uniformly distributed in the matrix. Cagrialp [27] modified basalt fibers with a silane coupling agent (3-aminopropyl). The study found that BF improves the tensile properties of composite materials, bending performance and impact resistance. Lee [28] used modifiers for surface treatment of basalt fiber. The study found that the chemically treated fibers improved the mechanical interfacial properties, interlaminar shear strength (ILSS) and fracture toughness (KIC) of the composites. H. Haido investigated the properties of basalt fiber SCC at high temperatures and formulated new constitutive models to simulate the real behavior of BFRC material under different load conditions. This numerical analysis was applied to BFRC beams with different fiber dosages and subjected to static loading at the whole range of applied load up to collapse. [29,30]. Basalt fiber is becoming more widely used in emulsified asphalt mixture [31,32,33]. The current research focuses on the application of modified basalt fiber in cement-based materials, composites, and hot-mix asphalt mixtures. Scholars have seldom studied the modification mechanism and effect of basalt fiber and the performance of modified fiber-enhanced emulsified asphalt evaporation residue. It is extremely important to study the enhancement mechanism of evaporation residue of basalt fiber emulsified asphalt.

In this paper, silane coupling agent was used for surface treatment of basalt fiber (BF) to improve the interface bonding and adhesion property between basalt fiber and emulsified asphalt evaporation residue. The effects of coupling-modified basalt fiber (MBF) on the high and low temperature rheological properties of emulsified asphalt evaporation residue were examined by three test methods of conventional testing: dynamic shear rheology, bending beam rheology, and force ductility. Elemental changes of fibers before and after modification and the microstructure of evaporation residue of coupling-modified fiber emulsified asphalt were analyzed. This paper considers the enhancement mechanism of coupling-modified fiber on the performance of emulsified asphalt evaporation residue, and provides theoretical reference and guidance for the popularization and application of fiber in emulsified asphalt cold recycling mixture.

## 2. Raw Materials

### 2.1. Emulsified Bitumen

The asphalt used throughout this paper is AH-70 matrix asphalt. The emulsifier used is white paste cationic emulsifier, the pH regulator used is hydrochloric acid, the stabilizer used is polyvinyl alcohol (PVA), and the anhydrous calcium chloride mass ratio is 1:1. The basic parameters of self-made cationic emulsified asphalt are given in Table 1. SBR latex was added by emulsification before modification; the basic parameters are given in Table 2.

### 2.2. Basalt Fiber

**Figure 1:**
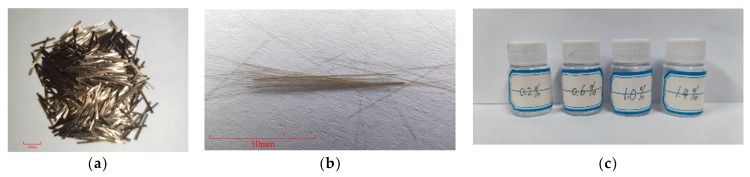
Basalt fibers and KH550 used in the study: (**a**) basalt fiber; (**b**) basalt fiber 5×; (**c**) KH550.

The fiber adopted was short-cut basalt fiber and its appearance is shown in Figure 1a. The performance index is given in Table 3. Basalt fibers need to be treated before being used. Basalt were placed in deionized water and stirred fully to ensure the dispersion of fibers and remove surface impurities. In order to remove the moisture and surface wetting agent on the fiber surface, the washed basalt fiber was dried at 120 °C for 1.5 h. The basalt fiber was weighed when it was completely dried to avoid the influence of surface impurities on the modification effect.

### 2.3. Silane Coupling Agents

The modification effect of silane coupling agent KH550 (molecular formula C9H23O3NSi, 3-aminopropyltriethoxysilane, Guobang Chemical Co., Ltd. Jinan, China) is the best [30]. In this paper, KH550 was selected as the modifier for the surface treatment of BF, as showed in Figure 1c. The performance indexes are presented in Table 4.

## 3. Experimental Details and Methods

### 3.1. Preparation of Silane Coupling Agent Modified Basalt Fiber (MBF)

**Figure 2:**
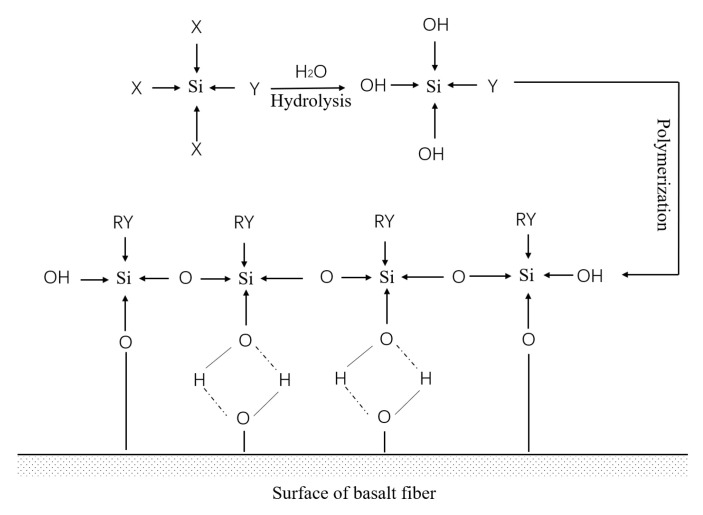
Flow chart of low temperature evaporation residue.

A mixture of anhydrous ethanol and distilled water with a 95:5 mass ratio was used to prepare organic solvents. The coupling modified solutions were 0.2%, 0.6%, 1.0%, and 1.4% KH550. Basalt fiber infiltration time was 10 min, 30 min, 60 min, and 90 min. After reaching the specified infiltration time, the treated BF was put into a 150 °C oven and dried, which made the silane coupling agent react fully with the surface of the BF to form a coupling agent layer. The reaction principle between KH550 and basalt fiber is shown in Figure 2. The ethoxy groups the silane coupling agent was transformed into silane alcohol groups through a hydrolysis reaction, and the silane alcohol groups was polymerized with O-H groups, resulting in the formation of covalent bonds on the fiber surface. The adjacent silanol groups solidified each other to form a dense silanol network structure [34,35,36].

### 3.2. Preparation of Emulsified Asphalt Evaporation Residue

**Figure 3:**
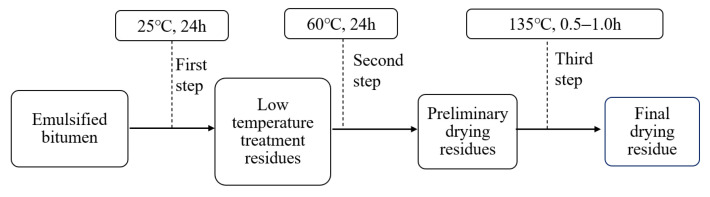
Flow chart of low temperature evaporation residue.

The evaporation residue of emulsified asphalt prepared by electric furnaces causes the aging of the evaporation residue, which easily affects test results. In order to reflect the demulsification process of emulsified asphalt [2], the evaporation residue of emulsified asphalt was prepared using the low-temperature evaporation method of ASTM D7497-09. The specific method is illustrated in Figure 3.

The modified basalt fiber with mass fractions of 0.5%, 1.0%, 1.5%, 2.0%, and 2.5% was added to the evaporation residue of emulsified asphalt. In order to prepare emulsified asphalt evaporation residues with different fiber contents, the high-speed shear was sheared at 1200 rpm for 15 min.

### 3.3. Test Details

**Figure 4:**
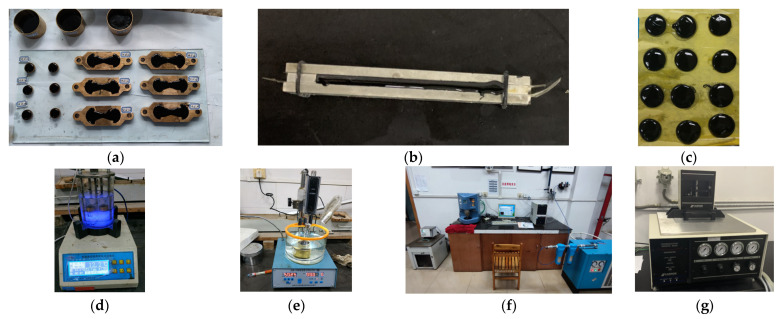
Performance test of emulsified asphalt residue: (**a**) specimens of conventional performance test; (**b**) specimens of bending beam rheological test (BBR); (**c**) specimens of dynamic shear rheometer (DSR) test; (**d**) softening point test; (**e**) penetration degree test; (**f**) dynamic shear rheometer (DSR) test; (**g**) bending beam rheological test (BBR).

(1) Penetration test: The specimens were placed in a 25 °C water bath for 1.5 h and each specimen was tested three times by 100 g load standard needle and 5 s penetration time. When the three parallel test results were within the allowable error range, the average value of the three test results was taken as the final test results.

(2) Softening point test: The specimens, steel balls, and a positioning ring were placed in 5 °C water bath environments for at least 15 min. Then, heating was started at 5 °C, with a heating rate of 5 °C ± 0.5 °C per minute until the two samples contacted with the bottom plate. The temperature of the specimen in contact with the floor was recorded, and the average value was considered as the final test result.

(3) Force-ductility test: The specimens were kept in a 15 °C water bath for 1.5 h and the tensile speed was 5 cm/min ± 0.25 cm/min. The readings were recorded when the specimen was fractured, and the average value of the three parallel experiments within the error range was taken as the ductility test result, in cm.

(4) Dynamic shear rheometer (DSR) test: The sample thickness was 1 mm; the diameter was 25 mm; the strain was controlled; the strain value was set to 12%; the frequency was 10 rad/s. A temperature scan was conducted from 46 to 76 °C, with a temperature step of 6 °C.

(5) Bending beam rheological test (BBR): The specimen size was 127 ± 2 mm long, 12.7 ± 0.05 mm high, 6.35 ± 0.05 mm wide; the test temperature was −12 °C and −18 °C; the test load was 980 ± 50 mN. Before the test, the specimens were placed in anhydrous ethanol at the test temperature for 1 h. Three parallel specimens were tested for each group of specimens and the average value was taken as the test result. The test specimen is shown in Figure 4.

### 3.4. Test Methods

(1) Performance test of silane coupling-modified fiber: In this paper, the coupling modification solutions with mass ratios of 0.2%, 0.6%, 1.0%, and 1.4% were selected to modify basalt fibers for 10 min, 30 min, 60 min, and 90 min. To test singer fiber tensile strength, basalt monofilaments were separated under a microscope. The drawing speed was 5 mm/min, the chuck gap was 25 mm, and the pre-tension was 0.2 cN. During the test, the fiber was guaranteed to break in the middle, and no less than 10 valid data are tested for each group of samples. The fracture strength and elongation at break of the basalt fiber were tested by YG001N electronic single fiber strength tester (Deshe Precision Instrument Co., Ltd. Changzhou, China) to determine the silane coupling modifier concentration and infiltration time.

(2) Performance tests of silane coupling-modified fiber emulsified asphalt: Performance was tested with the asphalt conventional performance test, the dynamic shear rheological (DSR) test, the bending beam rheological test (BBR), and the force ductility test (FDT), in order to study the effect of coupling-modified fiber (MBF) content (0.5%, 1.0%, 1.5%, 2.0%, 2.5%) on the high and low temperature rheological properties of emulsified asphalt evaporation residue.

(3) Microscopic test: In order to analyze the elemental changes of fiber before and after modification and the microstructure of fiber emulsified asphalt mortar, and to study the mechanism of silane coupling-modified fiber reinforced emulsified asphalt evaporation residue, scanning electron microscopy, energy spectrum analyzer, X-ray diffraction (XRD), and Fourier transform infrared spectroscopy were used.

## 4. Experimental Results and Discussions

### 4.1. Performance Analysis of Silane Coupling Agent-Modified Basalt Fiber (MBF)

#### 4.1.1. Breaking Strength and Breaking Elongation

**Figure 5:**
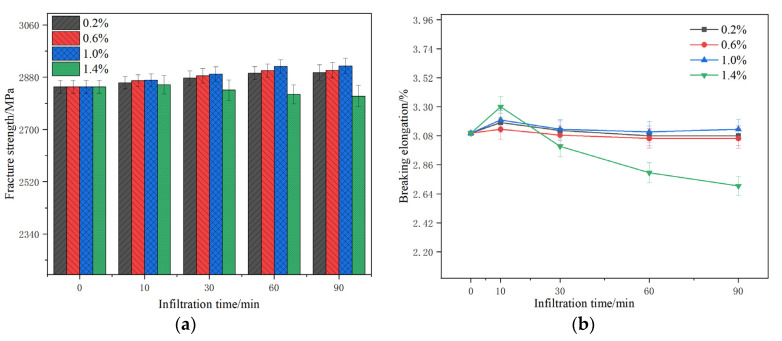
Effect of KH550 on basalt fiber properties: (**a**) fracture strength; (**b**) breaking elongation.

The impact of the silane coupling agent on the breaking strength and breaking elongation of basalt fiber filaments is shown in Figure 5.

Figure 5a is shown that for the same infiltration time, as the concentration of the KH550 solution increases, the breaking strength of the basalt fiber first increases and then decreases. When immersed for 60 min, the breaking strength of 1.0% basalt fiber increased from 2845 MPa to 2917 MPa. When the coupling agent concentration ranged from 0.2% to 1.0%, the breaking strength of the basalt fiber constantly increased with the increase of the infiltration time and was finally generally flat. Figure 5b demonstrates that when the infiltration time was 60 min, the elongation at break at 1.0% concentration was relatively high. With the extension of the soaking time, the elongation at break of the 1.0% concentration fiber constantly increased. The elongation at breaking of fibers of other concentrations decreased. The elongation at breaking at 1.4% concentration was reduced significantly, while the elongation at break at other concentrations did not change significantly. On the other hand, when the concentration of KH550 solution increased from 0% to 1.4%, the breaking strength dropped to 2821 MPa and the breaking elongation dropped to 2.8%. Excessive KH550 concentration will degrade the breaking strength of the fiber and decrease its elongation at break.

In summary, optimum silane coupling agent concentration was 1.0% and the optimum infiltration time was 60 min. The effect of silane coupling-modified fibers on the properties of evaporated residues of emulsified asphalt was subsequently investigated.

#### 4.1.2. Microscopic Analysis of Silane Coupling Agent Modified Basalt Fibers (MBF)

(1) Scanning electron microscopy (SEM)

**Figure 6:**
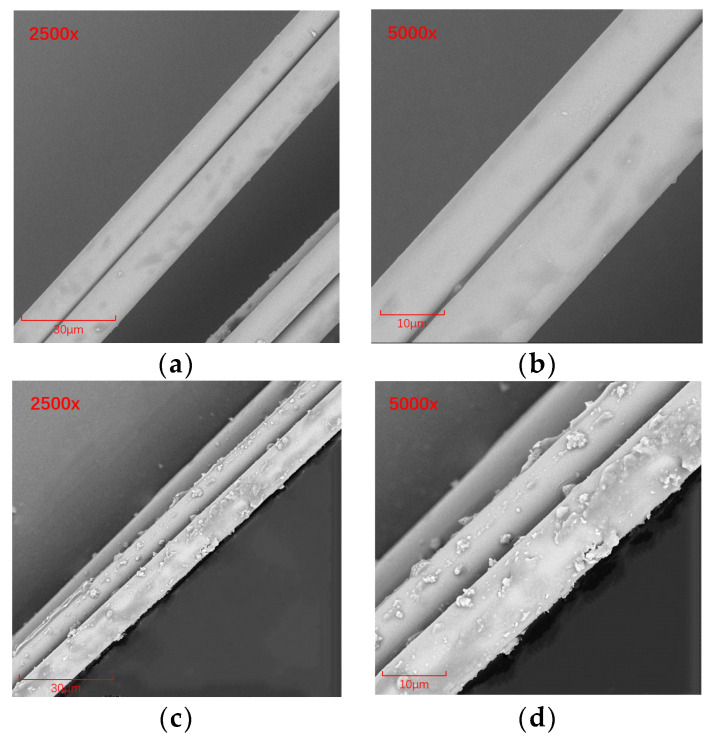
SEM images of basalt fiber before and after modification: (**a**) BF pretreated by 2500×; (**b**) 5000× pretreatment BF; (**c**) 2500× modified BF; (**d**) 50000× modified BF.

The original samples of BF and MBF were gold sprayed and the modification effect on the fiber surface was noted using scanning electron microscopy. The microscopic morphology is illustrated in Figure 6. 

Figure 6 demonstrates that the original sample basalt fibers have a smooth surface and no surface protrusions. The bonding performance with the emulsified asphalt base is insignificant. It easily causes adverse effects, such as sliding out and flaking, and cannot play the role of “bridging” to toughen the fibers [37,38]. Modified basalt fibers have blocky, stripy, and scale-like projections. This indicates that the silane coupling agent polymerized with the fiber surface, increasing its surface roughness. The silane film also further avoids contact with external corrosive media, which enhances the strength and chemical stability of the fibers themselves. At the same time, the silane film enhances the bite between the fibers and the emulsified asphalt, making it less likely to come off as a whole when fractured. This will improve the friction effect between the basalt fiber and the asphalt mixture and improve the mechanical properties of the mixture.

(2) X-ray energy spectrometer (EDS)

**Figure 7:**
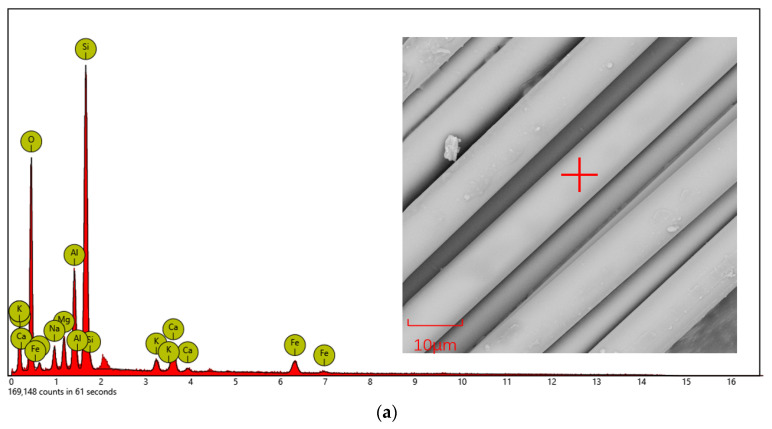

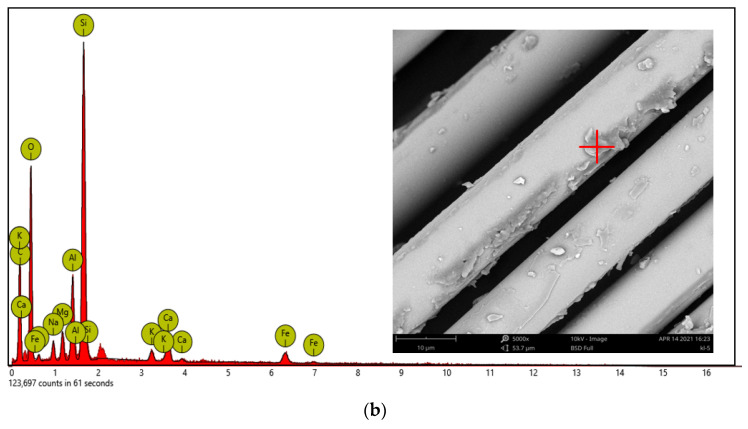
EDS composition analysis of basalt fiber before and after modification: (**a**) original BF; (**b**) coupling modification BF.

The elemental composition was analyzed by X-ray spectrometry and the elemental changes before and after the coupling modification are shown in Figure 7 and Table 5.

Figure 7 and Table 5 show that the basalt fibers are mainly composed of O, C, Si, and Al, and also include small amounts of Na, Fe, and Ca. Comparing the fibers before and after modification, the atomic percentage of O decreased from 47.56 to 33.47 and the atomic percentage of Si increased from 7.78 to 10.71. The analysis indicated that the silane coupling agent generated Si–O chemical bonds within the fiber matrix [35], which caused the change of the O to Si ratio. This translated into the appearance of blocky and scale-like projections on the fiber surface, which enhanced the roughness of the fiber. This feature improves the interfacial bonding ability of the fibers to the emulsified asphalt matrix.

(3) X-ray diffractometer (XRD)

**Figure 8:**
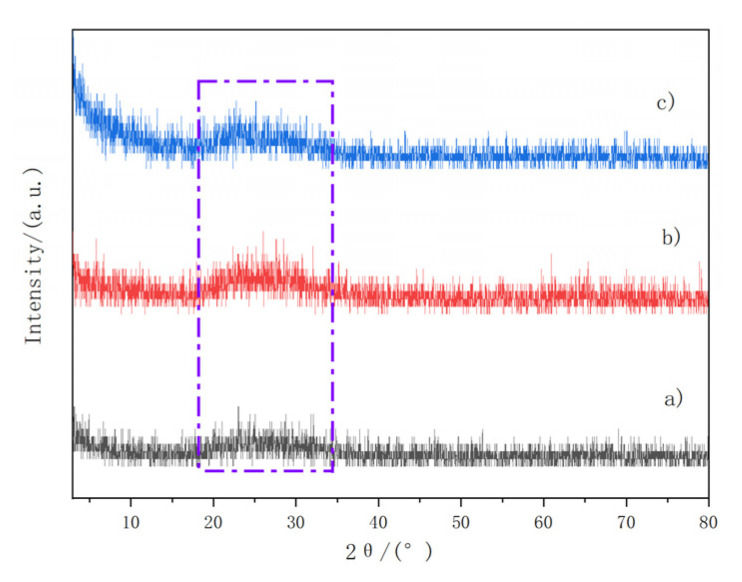
XRD patterns of basalt fiber before and after modification: (**a**) original fiber; (**b**) pretreated fibers; (**c**) coupling-modified fibers.

X-ray diffraction technology (XRD) was used to analyze the crystal structure of the original fiber and pretreated fiber, the optimal treatment time, and the concentration-modified fiber. The scanning angle was 3° to 80° and the scanning speed was 10(°)/min. The test results are shown in Figure 8.

Figure 8 shows that the XRD ridges of the original fiber, pretreated fiber, and coupling-modified fiber are very close. There were no significant differences in crystallization before and after the coupling modification. The XRD analysis presented an amorphous diffraction pattern with weak diffraction peaks and low crystallinity, which are characteristic of amorphous diffraction patterns [39]. In the process of fiber modification, the appearance of crystal structure becomes the weak point of the interface and degrades the breaking strength of the fiber [40,41]. The XRD pattern shows that the product of the polymerization reaction of the silane coupling agent is not causing the fiber to produce a crystal structure.

(4) Fourier transform infrared spectroscopy (FT-IR)

In order to further analyze the functional group changes of silane coupling agent-modified fiber, Fourier transform infrared spectroscopy (FT-IR) was used to study the modification effect.

**Figure 9:**
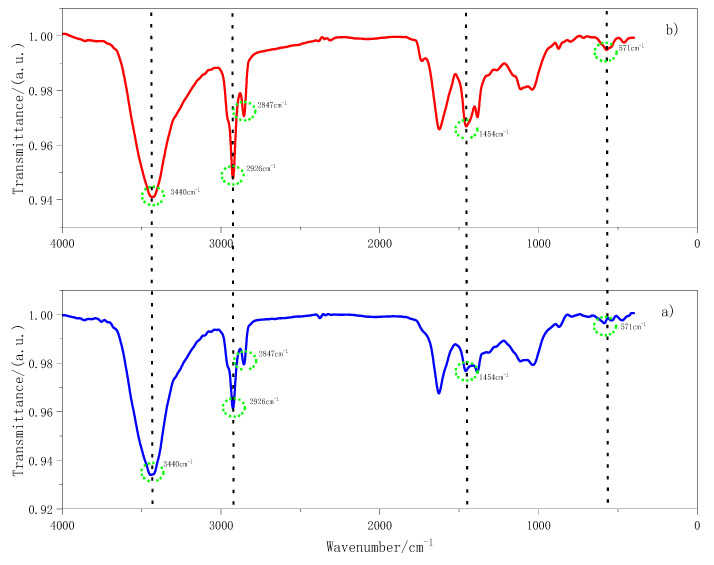
Infrared spectra of basalt fiber before and after modification: (**a**) BF pretreatment; (**b**) coupling-modified BF.

Figure 9 demonstrates the main absorption peaks of basalt fiber functional groups: the (-OH) characteristic peak at 3440 cm^−1^, the (-CH) characteristic peak at 2926 cm^−1^ and 2847 cm^−1^, and the benzene ring at 1620 cm^−1^, along with the characteristic peak of yoke double bond (C=C), the partial (-CH3) asymmetric deformation vibration at 1454 cm^−1^, and the partial methylene (-CH2) shear vibration characteristic peak with the (Si–O) peak at 574 cm^−1^), etc. The changes in the absorption peaks of functional groups show that the changes in absorption peaks are mainly concentrated in functional groups such as (-OH), (-CH2), (-CH3), and (Si–O). Compared with the pretreatment BF, the characteristic peak of (-OH) of MBF is obviously reduced, and the characteristic peak of the Si–O group is slightly increased, which indicates that the (-OH) groups on the surface of the BF and the silanol groups of KH550 have polymerized. At the same time, adjacent silanol groups are cured with each other, so that the Si–O groups are further increased. This will form a dense silane network structure to improve the fiber surface roughness.

### 4.2. Performance Analyses of Emulsified Asphalt Evaporative Residues Reinforced by MBF

#### 4.2.1. Effect of MBF Content on the Performance of Emulsified Asphalt Evaporation Residue

I. Conventional Performance Test

(1) Injectivity and softening point

**Figure 10:**
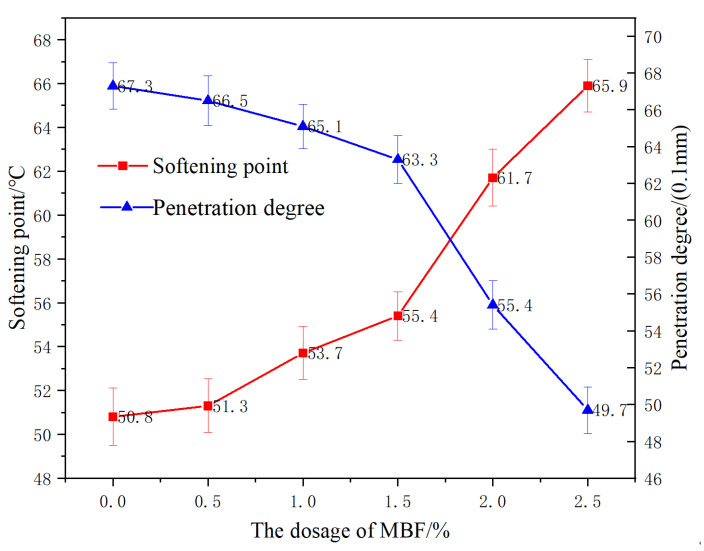
Effect of coupling-modified fiber content on the penetration and softening point of evaporative residue.

The effect of MBF on the penetration and softening point of evaporation residue of matrix-emulsified asphalt is illustrated in Figure 10.

Figure 10 demonstrates that the content of MBF increased from 0% to 2.5%. Needle penetration of the evaporated residue of the matrix-emulsified asphalt decreased from 67.3 (0.1 mm) to 49.7 (0.1 mm). The softening point increased from 50.8 °C to 65.9 °C, with a percentage increase of 29.7%. Due to the addition of MBF, the consistency of the evaporated residue increased, which restricted the free flow of asphalt and thus led to a decrease in the needle penetration. At the same time, the surface area of MBF is relatively enormous, and the adsorption effect on asphalt is produced after it is mixed into the evaporated residue of emulsified asphalt, making it form a stable structure asphalt film, which improves the softening point and high temperature stability.

(2) Ductility

**Figure 11:**
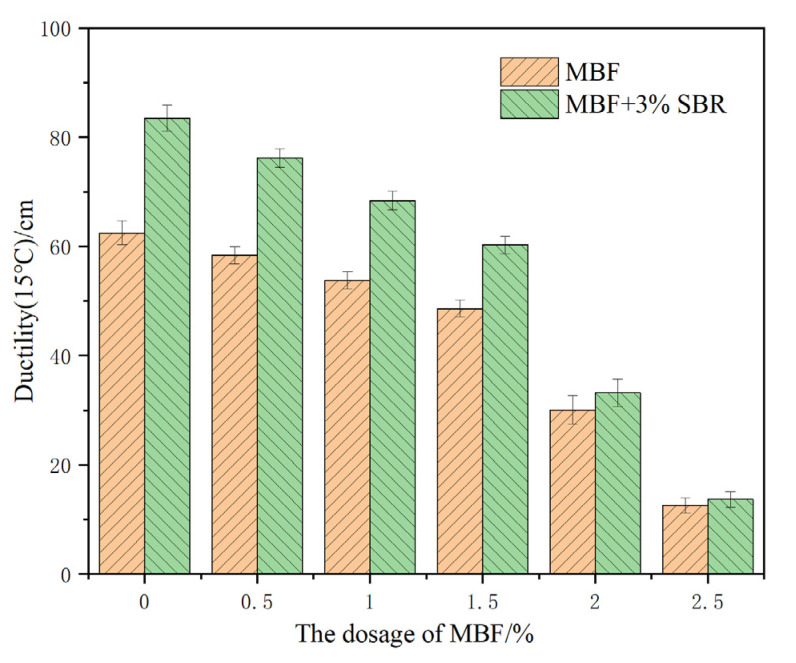
Effect of MBF on the 15 °C ductility of evaporation residue.

The effect of MBF on the 15 °C ductility of evaporation residue of matrix-emulsified asphalt and SBR emulsified asphalt is shown in Figure 11.

Figure 11 demonstrates that the percentage of MBF increased from 0% to 2.5%. The ductility of the emulsified asphalt evaporated residue with MBF decreased significantly from 63.5 cm to 12.6 cm. When the MBF dose was greater than 1.5%, the ductility decreased sharply. 3% SBR latex could dramatically improve the ductility of the emulsified asphalt evaporated residue. With the increase of the proportion of MBF, the SBR improvement effect gradually decreases. The analysis found that when the proportion of MBF was greater than a specific value, the dispersion effect of MBF in the evaporated residue became worse. The phenomenon of agglomeration, and thus stress concentration, easily occurs, resulting in the reduction of elasticity. This suggests that the proportion of MBF should not be too large.

II. Dynamic Shear Rheology Test

In order to further investigate the optimal dosing of MBF, the high and low-temperature rheological properties of MBF emulsified asphalt evaporated residues were evaluated. The dynamic shear rheological test parameters were: strain control mode; strain control of 12%; frequency of 10 rad/s; temperature range of 46 °C−76 °C; temperature interval of 6 °C; G*/sinδ ≤ 1.0 kpa after stopping.

(1) Phase angle and complex shear modulus

**Figure 12:**
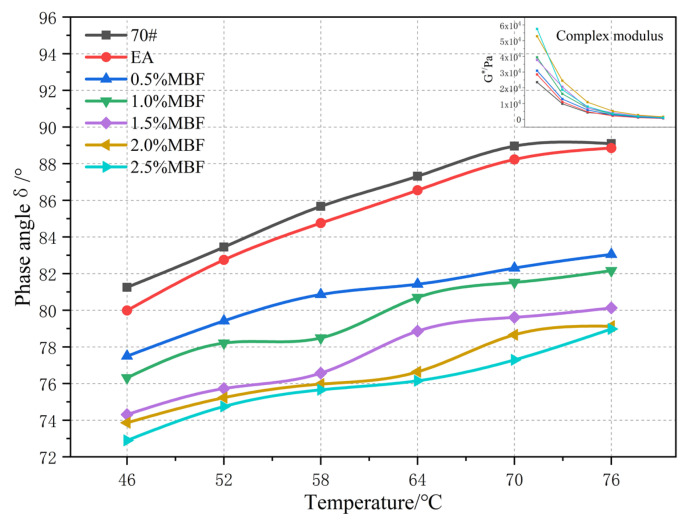
Effect of MBF on δ and G* of emulsified asphalt evaporation residue.

The phase angle δ and complex modulus G* of MBF emulsified asphalt evaporation residue at different temperatures are shown in Figure 12.

Figure 12 shows that with the increase of temperature, the phase angle δ of MBF emulsified asphalt evaporation residue increases. The complex modulus G* moderately decreases, and the decrease tends to be flat. In the high-temperature part, the index of evaporation residue of MBF emulsified asphalt is similar to that of No. 70 matrix asphalt. The analysis demonstrates that the viscosity component in emulsified asphalt increases with the increase of temperature. The elastic composition of evaporation residue of MBF emulsified asphalt is relatively reduced, as it has similar temperature sensitivity to matrix asphalt. At the same temperature, MBF greatly reduces the phase angle δ of emulsified asphalt and increases the complex modulus G*. The viscosity composition of emulsified asphalt matrix increases, which improves the high temperature performance of emulsified asphalt and enhances the matrix.

(2) Rutting factor

**Figure 13:**
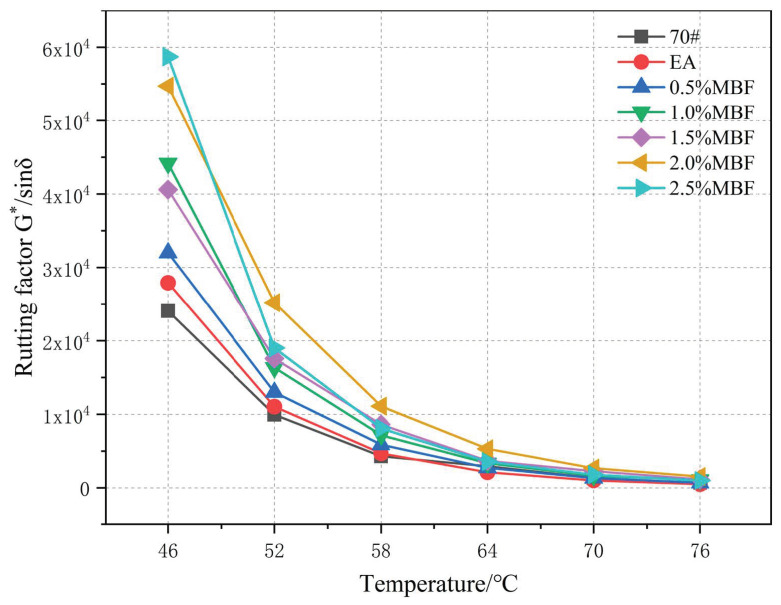
Effect of MBF on G*/sinδ of emulsified asphalt evaporation residue.

Figure 13 shows that the rutting factor of evaporation residue of MBF emulsified asphalt gradually decreases with the increase of temperature. After the temperature exceeds 58 °C, the decline gradually becomes gentle. With the increase of temperature, the high-temperature anti-rutting effect of MBF slightly decreases. This shows that the evaporation residue of MBF emulsified asphalt has obvious temperature sensitivity. At the same temperature, the rutting factor increases with the increase of the content of the coupling-modified fiber. When the temperature reaches 58 °C, the ratio of MBF increases from 0 to 1.5%, and G*/sinδ increases by 1.3 kPa. It shows that MBF can enhance the high-temperature rutting resistance of emulsified asphalt. Analysis suggests that the specific surface area of MBF can absorb the light components in the emulsified asphalt evaporation residue. At the same time, the surface of MBF is rough, which increases the mechanical engagement ability with asphalt when forming a fiber-interleaved structure. MBF reduces the fluidity of the emulsified asphalt evaporation residue and improves the high-temperature rutting resistance.

III. Low-Temperature Performance

The low-temperature performance of emulsified asphalt evaporation residue is closely linked to the low-temperature crack resistance of cold recycled mixture. The effect of modified fiber on the low-temperature rheological properties of emulsified asphalt was studied by the force ductility test (FDT) and bending beam rheological test (BBR).

(1) Force ductility test (FDT)

**Figure 14:**
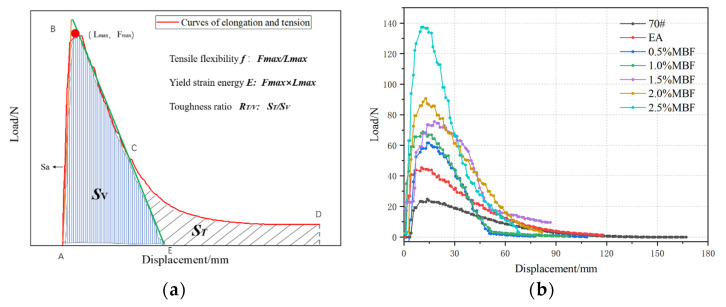
Force ductility diagram of coupling-modified BF evaporation residue at 5 °C: (**a**) tension—ductility indicator diagram; (**b**) tensile—elongation force curve.

Figure 14b shows that when MBF is added to the emulsified asphalt evaporation residue, the maximum tensile force corresponding to the ductility at 5 °C continuously increases. The maximum tensile force is 138.7 N. Comparing with the base emulsified asphalt, the increase rate reaches 206%. This shows that during the stretching stage, the distribution of MBF in the emulsified asphalt is relatively uniform. The site effect of MBF on the emulsified asphalt leads to an increase in the maximum tensile force. MBF can greatly improve the shear resistance of the emulsified asphalt matrix. In order to fully evaluate the effect of MBF on the low-temperature rheological properties of emulsified asphalt matrix, tensile flexibility *f: F_max_/L_max_*, yield strain energy *E: F_max_* × *L_max_*, and toughness ratio *R_T/V_: S_T_/S_V_* indexes are introduced. The calculation formula and schematic diagram of the corresponding parameters is shown in Figure 14a. Slope Sa of the AB section straight line is the stiffness modulus of the test piece. Figure 14b shows that with the increase of MBF content, the stiffness modulus is continuously increases. The ductility value drops rapidly, and the yield strain energy increases. MBF deteriorates the low-temperature performance of emulsified asphalt.

(2) Toughness ratio *R_T/V_*: *S_T_/S_V_*

**Figure 15:**
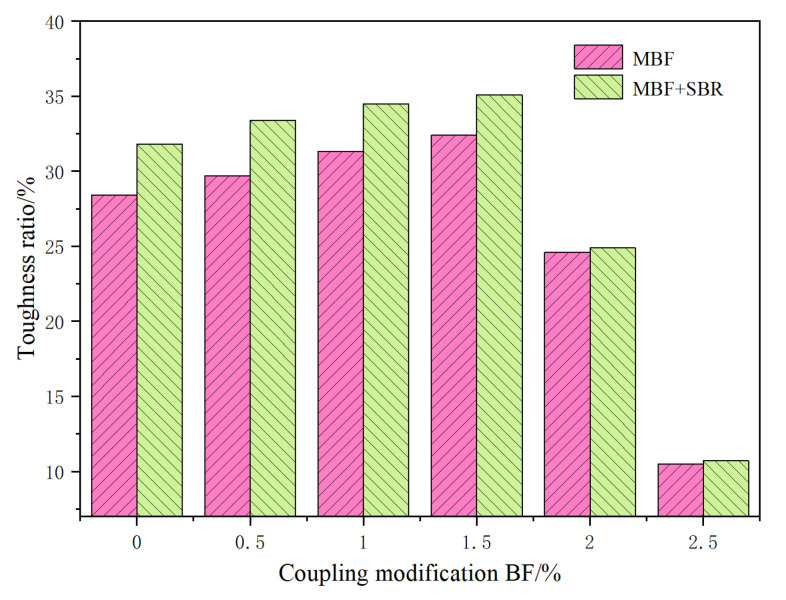
Toughness ratio *R_T/V_* diagram of coupling-modified BF evaporation residue.

Figure 15 shows that with the increase of MBF content, the *R_T/V_* index first increased and then decreased. *R_T/V_* and MBF doping have a highly nonlinear relationship. When the MBF content is 1.5%, the toughness ratio index is the greatest. SBR has good viscoelasticity. Adding SBR latex increases the toughness ratio of the evaporation residue of MBF emulsified asphalt. However, with the increase of MBF content, the improvement effect is gradually reduced. Analysis suggests that when the amount of MBF is small, MBF can absorb the emulsified asphalt evaporation residue. This process will form a stable structure asphalt membrane and enhance the mechanical properties of emulsified asphalt. MBF has little effect on the elastic properties of emulsified asphalt evaporation residue. When the MBF content exceeds 1.5%, the dispersibility of MBF in the emulsified asphalt evaporation residue is poor. Although the tensile force increases, stress is concentrated and weak interface points appear, which leads to a substantial decrease in its ductility. Adding SBR can improve the low-temperature ductility of the evaporation residue of MBF emulsified asphalt, but the content of MBF should not exceed 1.5%.

(3) Bending beam rheological test (BBR)

The low-temperature creep properties of emulsified asphalt evaporation residue with different MBF content were analyzed by SYD-0627 bending beam rheometer (Deshe Precision Instrument Co., Ltd. Changzhou, China). The test temperatures were −12 °C and −18 °C. The specimen size was 127 ± 2 mm long, 12.7 ± 0.05 mm high, 6.35 ± 0.05 mm wide, and the load was 980 ± 50 mN. The test results are presented in Table 6.

Table 6 shows that at the same temperature, with the increase of MBF content, the creep stiffness S continuously increases. When the MBF content is low, the initial creep stiffness increases slowly. When the MBF content exceeds 1.5%, the creep stiffness increases faster. Under the condition of −12 °C, the proportion of MBF increased from 0.5% to 2.5%, and the creep stiffness of the evaporation residue of MBF emulsified asphalt increased by 7%, 18%, 24.8%, 57.1%, and 78.1%. At 18 °C, the creep stiffness of the evaporation residue of MBF emulsified asphalt is greater than 300 Mpa.

Adding SBR latex will restore part of the low-temperature creep properties. When the MBF content exceeds 1.5%, the creep rate m of the emulsified asphalt evaporation residue is markedly reduced. Under the condition of −12 °C, the creep rate m of 1.5% MBF compounded SBR latex increased from 0.331 to 0.342. Analysis suggests that MBF can be adsorbed with the emulsified asphalt matrix, which will produce a more durable structured asphalt membrane and increase the interfacial bonding force. Because MBF has the characteristics of high modulus and high tensile performance, it has a weak influence on the low-temperature performance of the emulsified asphalt evaporation residue. Nevertheless, when the amount of MBF is too much, MBF will cluster inside the matrix, which will adversely affect the low-temperature crack resistance of the emulsified asphalt matrix. Adding SBR latex will improve the low-temperature creep performance of the emulsified asphalt evaporation residue. The recommended amount of MBF is 1.5%.

#### 4.2.2. Mechanism Analysis of MBF Emulsified Asphalt Evaporation Residue

I. Principle of SBR-Enhanced Ductility of Emulsified Asphalt

**Figure 16:**
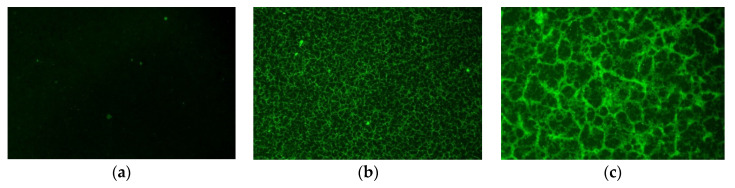
SBR-emulsified asphalt fluorescence microscope: (**a**) matrix-emulsified asphalt; (**b**) 100× magnification 3% SBR; (**c**) 400× magnification 3% SBR.

The distribution of SBR latex in emulsified asphalt was observed by XDY-1 fluorescence microscope. The experimental results are presented in Figure 16.

Figure 16 shows that the matrix-emulsified asphalt does not show fluorescence characteristics under the fluorescence irradiation of a mercury lamp. Emulsified asphalt is continuous and dark green. Some fluorescent points are emulsifiers. When 3% SBR latex is mixed with emulsified asphalt, a three-dimensional network structure is formed. The network structure can improve the ductility and flexibility of the matrix. The deformation resistance of the emulsified asphalt matrix is greatly improved. SBR emulsion is uniformly distributed in the emulsified asphalt matrix without agglomeration. SBR emulsion has excellent compatibility with emulsified asphalt, which enhances the low-temperature crack resistance of emulsified asphalt. The microstructure is in agreement with the macroscopic performance.

II. The Effect of MBF on Functional Groups of Emulsified Asphalt

**Figure 17:**
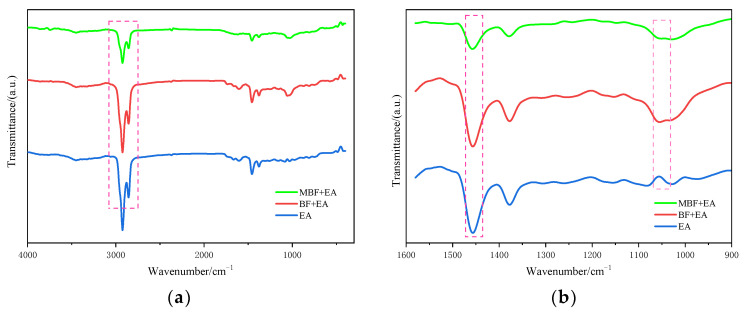
Infrared spectrum of fiber-emulsified asphalt before and after modification: (**a**) full band; (**b**) 900 cm^−1^–1600 cm^−1^ band.

The adsorption of functional groups on emulsified asphalt by MBF was investigated by Fourier transform infrared spectroscopy. The effect of MBF on the position and number of functional groups of emulsified asphalt was analyzed to study the mechanism of fiber increasing the adhesion of emulsified asphalt. The matrix-emulsified asphalt (EA), emulsified asphalt adsorbed by 1.5% basalt fiber (BF + EA), and emulsified asphalt adsorbed by 1.5% MBF (MBF + EA) were used for the infrared spectroscopy test. The test results are presented in Figure 17.

Figure 17 shows that matrix-emulsified asphalt EA, BF + EA, and MBF + EA have relatively prominent peaks at 2926 cm ^−1^, 2847 cm^−1^, 1462 cm^−1^, and 1034 cm^−1^. 2926 cm^−1^ is the symmetric and asymmetric vibration of methylene (-CH2). 1462 cm^−1^ is the shear vibration of (-CH2-) and the symmetric vibration of (-CH3). The absorption peak of 1034 cm^−1^ is caused by (S=O) stretching vibration. The three emulsified asphalt peak positions are consistent, but the size of the absorption peak is different. BF emulsified asphalt in 2926 cm^−1^, 2847 cm^−1^, and 1462 cm^−1^ peaks has different degrees of weakening. Nonetheless the magnitude changed little. The peak of MBF emulsified asphalt reduced significantly at the same position. It indicates that MBF can better adsorb the manageable components and unsaturated hydrocarbon chains in emulsified asphalt and has a better stability effect on emulsified asphalt.

III. Microstructure Analysis of MBF-Emulsified Asphalt

**Figure 18:**
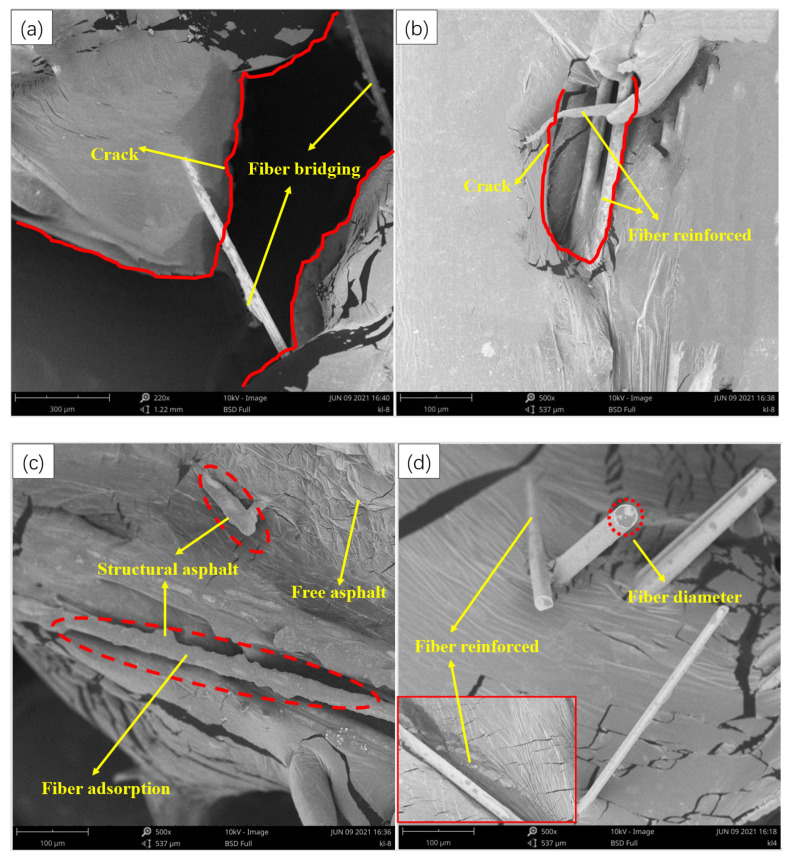

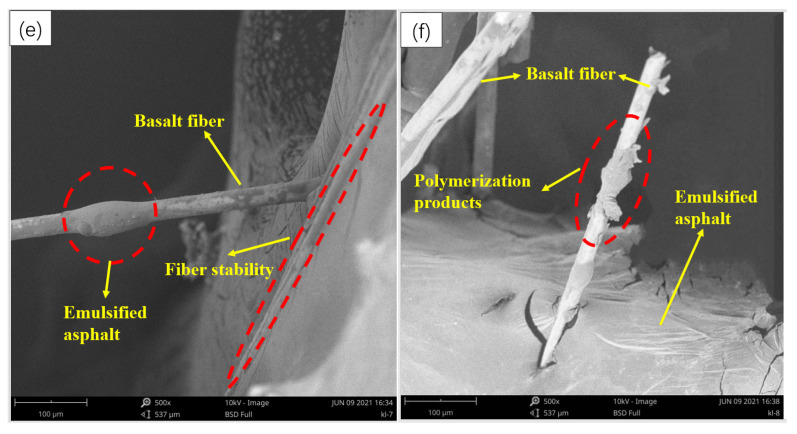
Microstructure of fiber-emulsified asphalt: (**a**) fiber bridging effect; (**b**) fiber stabilization effect; (**c**) fiber adsorption effect; (**d**) fiber reinforcement effect; (**e**) latex bead; (**f**) modified fiber polymerization product.

To further study the distribution and strengthening mechanism of the MBF system in emulsified asphalt evaporation residue, the evaporation residue of emulsified asphalt with 1.5% MBF content was observed by scanning electron microscopy (SEM). The distribution of MBF in emulsified asphalt is shown in Figure 18.

Figure 18 shows that the distribution of MBF in emulsified asphalt is mostly monofilament. MBF does not display a large-scale clustering phenomenon and has good dispersion uniformity.

Figure 18a,b show that MBF plays a “bridging” role in the emulsified asphalt matrix. When the emulsified asphalt matrix is subjected to external stress and cracks, the fiber can block the development of cracks due to the high modulus and high tensile strength, thereby improving the crack resistance of the emulsified asphalt matrix.

Figure 18c,d show that the fibers are intricately allocated in the emulsified asphalt matrix and interspersed with each other. The fibers can constitute a three-dimensional network structure by overlapping, which plays a reinforcing role. Because MBF is a flexible material, it can improve the internal friction angle of the emulsified asphalt mixture. At the same time, the flow of loose asphalt is restricted and the stabilizing effect of the emulsified asphalt matrix is enhanced.

Figure 18e,f show that the surface of MBF is relatively rough, with scaly and massive bumps, which can be better adsorb asphalt. The theory of interface mechanics suggests that the combination of reinforced fiber and asphalt matrix can prevent MBF from breaking, falling off, and peeling off, which can enhance the mechanical properties of emulsified asphalt. A thick asphalt structure film is established on the surface of the MBF, which enhances the adhesion effect with the emulsified asphalt matrix. This makes the asphalt matrix maintain better temperature stability at high temperatures, improving the modulus of the emulsified asphalt matrix and enhancing the ability of the emulsified asphalt matrix to resist permanent deformation.

## 5. Conclusions

(1) The best treatment concentration of KH550 is 1.0% and the best infiltration time is 60 min. Microscopic analysis and functional group changes show that the surface of the silane coupling-modified fiber becomes rough, forming Si–O bonds, and the (-OH) characteristic peak is decreased, which enhances cohesion with the emulsified asphalt base.

(2) As the content of silane coupling-modified fiber increases, the high-temperature rheological properties of the emulsified asphalt evaporation residue greatly improve. There was a certain degree of degradation of the low-temperature rheological properties, but this influence is relatively small. A respectable amount of SBR latex can improve the low-temperature performance of the silane coupling-modified fiber emulsified asphalt.

(3) The toughness ratio *R_T/V_* index is introduced through the force measurement ductility test. When the MBF content is 1.5%, the toughness ratio index is the largest, reaching 32.5%. Considering the effect of MBF on the high- and low-temperature rheological properties of the emulsified asphalt evaporation residue, the recommended content of silane coupling-modified fiber (MBF) is 1.5%.

(4) From the perspective of microstructure, the mechanism of silane coupling-modified fiber’s stabilization, crack resistance, reinforcement, and bridging of emulsified asphalt evaporation residue is explained.

This paper proposes a method to enhance the interfacial bonding ability of fiber-emulsified asphalt. It provides theoretical reference and guidance for the popularization and application of silane coupling-modified fiber in emulsified asphalt cold recycled mixture.

## Figures and Tables

**Table 1:** Parameters of emulsified asphalt.

Fundamental Index		Specification Limits	Test Results
Particle charge		-	Cationic (+)
Evaporation residue	Evaporation residue content (%)	≥60	64.5
	Degree of postpone (15 °C)/cm	≥40	62.5
	Degree of needle (25 °C)/0.1 mm	50–130 mm	78.7
	Soften point	-	52
Storage stability	1d (%)	≤1	0.6
	5d (%)	≤5	2.4

**Table 2:** Properties of epoxy resin SBR.

Fundamental Index	Test Results
Appearance	Milky white liquid
Evaporation residue content/%	50 ± 1
Viscosity (mPa·s)	1300–2000
pH	5–7

**Table 3:** Properties of basalt fiber.

Fundamental Index	Test Results
Equivalent diameter/μm	13–16
Fiber length (mm)	10–12
Relative density (g/cm^3^)	2.4 ± 0.1
Breaking strength (MPa)	2800–3200
Elastic modulus (GPa)	80–90
Breaking elongation (%)	3.1
Heat resistance	170 ± 5 °C No change in surface

**Table 4:** Properties of KH550.

Fundamental Index	Test Results
Boiling point (°C)	213
Density (ρ^20^g/m)	0.949
Refractive index (ND25)	1.429
Flash point (°C)	88
Purity (%)	≥99

**Table 5:** Surface atomic number of basalt fiber before and after modification.

Element		O	C	Si	Al	Na	Fe	Mg	Ca
Original BF	Weight percentage	44.14	23.63	13.63	5.85	2.07	4.72	1.96	2.92
	Atomic percentage	47.56	34.57	7.78	3.81	1.58	1.48	1.41	1.28
Modification BF	Weight percentage	36.34	37.08	17.11	3.88	1.42	3.31	1.42	2.16
	Atomic percentage	33.47	49.47	10.71	2.3	0.99	0.95	0.94	0.86

**Table 6:** Bending rheological parameters of MBF emulsified asphalt.

MBF		0%	0.5%	1.0%	1.5%	2.0%	2.5%	1.5% + SBR
−12 °C	S/MPa	133	143	157	166	209	247	153
	m	0.375	0.359	0.343	0.331	0.302	0.278	0.342
	*K*	354.66	398.32	457.72	501.51	692.05	888.48	418.12
−18 °C	S/MPa	347	369	394	414	465	513	407
	m	0.327	0.316	0.304	0.291	0.262	0.236	0.292
	*K*	1061.16	1167.72	1296.0	1422.68	1774.80	2173.72	1393.83

## Data Availability

The data used to support the findings of this study are included within the article.
